# Annexin A2: The Importance of Being Redox Sensitive

**DOI:** 10.3390/ijms14023568

**Published:** 2013-02-07

**Authors:** Patrícia A. Madureira, David M. Waisman

**Affiliations:** 1 Centre for Molecular and Structural Biomedicine, University of Algarve, Campus of Gambelas, Faro, 8005-139, Portugal; E-Mail: pamadureira@ualg.pt; 2 Departments of Biochemistry & Molecular Biology and Pathology, Dalhousie University, 5850 College Street, Halifax, Nova Scotia, B3H 4R2, Canada

**Keywords:** redox, reactive oxygen species (ROS), annexin A2, tumourigenesis, hydrogen peroxide (H_2_O_2_), reactive cysteine, glutathionylation, thiolate anion

## Abstract

Hydrogen peroxide (H_2_O_2_) is an important second messenger in cellular signal transduction. H_2_O_2_-dependent signalling regulates many cellular processes, such as proliferation, differentiation, migration and apoptosis. Nevertheless, H_2_O_2_ is an oxidant and a major contributor to DNA damage, protein oxidation and lipid peroxidation, which can ultimately result in cell death and/or tumourigenesis. For this reason, cells have developed complex antioxidant systems to scavenge ROS. Recently, our laboratory identified the protein, annexin A2, as a novel cellular redox regulatory protein. Annexin A2 possesses a reactive cysteine residue (Cys-8) that is readily oxidized by H_2_O_2_ and subsequently reduced by the thioredoxin system, thereby enabling annexin A2 to participate in multiple redox cycles. Thus, a single molecule of annexin A2 can inactivate several molecules of H_2_O_2_. In this report, we will review the studies detailing the reactivity of annexin A2 thiols and the importance of these reactive cysteine(s) in regulating annexin A2 structure and function. We will also focus on the recent reports that establish novel functions for annexin A2, namely as a protein reductase and as a cellular redox regulatory protein. We will further discuss the importance of annexin A2 redox regulatory function in disease, with a particular focus on tumour progression.

## 1. Introduction

Reactive oxygen species (ROS) are oxygen-containing reactive chemical species, which include biologically important molecules, such as superoxide (O_2_^•−^), hydroxyl radical (·OH) and hydrogen peroxide (H_2_O_2_). Until recently, ROS were regarded as harmful by-products of mitochondrial respiration, but it is now apparent that the ROS molecule, H_2_O_2_, is an important second messenger in cellular signal transduction. H_2_O_2_ levels are increased by various signalling proteins, including growth factors, cytokines, hormones and neurotransmitters through the activation of NADPH oxidase (Nox). The increased levels of H_2_O_2_ result in the targeting and oxidation of a relatively low abundance cysteine thiol residue, called a reactive cysteine residue or thiolate anion. The oxidation of these reactive cysteine residues, present in only a selected group of proteins, results in conformational changes and thereby regulates the function of the target molecule. Currently, H_2_O_2_-dependent signalling has been implicated in diverse processes, such as in the regulation of cell proliferation, differentiation, migration and apoptosis [1–3]. The production of a cytotoxic molecule has obvious potential risks to cells, as H_2_O_2_ is the major contributor to DNA damage, protein oxidation and lipid peroxidation that ultimately can result in cell death and/or tumourigenesis [4,5]. Cells have developed complex antioxidant systems to scavenge ROS. These include proteins, such as catalase, superoxide dismutases, glutathione peroxidases and thioredoxin peroxidases. The primary systems that reduce/recycle these ROS scavenging proteins are the thioredoxin (Trx) and the glutathione (GSH) systems.

Several studies have shown an important role for ROS in tumour development [6,7]. Cancer cells typically exhibit increased ROS generation compared to normal cell counterparts that not only provide cancer cells a proliferative advantage, but also promote malignant progression. In order to balance the proliferative advantage of ROS upregulation *versus* its potential risk due to protein, lipid and DNA damage, cancer cells induce the overexpression and/or activation of the cellular antioxidant systems.

Recently, our laboratory identified a novel cellular redox regulatory protein, called annexin A2, which interacts directly with H_2_O_2_ molecules in a reversible manner. Annexin A2 possesses an *N*-terminal reactive cysteine residue (Cys-8) that is readily oxidized by H_2_O_2_ and subsequently reduced by the thioredoxin system, enabling annexin A2 to participate in multiple redox cycles. Thus, a single molecule of annexin A2 can inactivate several molecules of H_2_O_2_. In summary, we showed that annexin A2 played a key role in cellular redox regulation, particularly during oxidative stress and tumourigenesis [8].

We will critically review the studies detailing the reactivity of annexin A2 thiols and the importance of these reactive cysteine(s) in regulating annexin A2 structure and function. We will also focus on the recent reports that establish novel functions for annexin A2, namely as a protein reductase and as a cellular redox regulatory protein. We will further discuss the importance of annexin A2 redox regulatory function in disease, with a particular focus on tumour progression.

## 2. Introduction to Reactive Oxygen Species

Reactive oxygen species (ROS), which include superoxide anion (O_2_^•−^), hydrogen peroxide (H_2_O_2_) and the hydroxyl radical (·OH) exist mainly at low concentrations in the cell (from 10^−4^ to 10^−9^ M) and are produced as by-products of normal aerobic metabolism by the mitochondria or in response to the activation of certain oxidases, such as NADPH oxidase and xanthine oxidase. These three ROS are linked by spontaneous or enzymatic dismutation [9]. Among the different ROS, ·OH is probably the most unstable with a half-life of 10^−9^ seconds. Furthermore, ·OH has no specificity, as it reacts rapidly with almost any organic molecule. The lifetime of O_2_^•−^ is also very short. O_2_^•−^ can oxidize thiols to thiyl radical, but this reaction is too slow to be physically relevant. Although it was originally suggested that O_2_^•−^ might play a role in cellular signalling, it is now thought that the signalling role of O_2_^•−^ is more likely that of functioning as a precursor of H_2_O_2_. H_2_O_2_ is more stable, but it is rapidly metabolized either enzymatically by catalase and GSH peroxidase or non-enzymatically by low concentrations of transitional metals, such as iron. Thus, the levels of H_2_O_2_ in the cell are dependent on the concentration of the detoxification enzymes and the rate of H_2_O_2_ formation. The high diffusibility of H_2_O_2_ and its ability to target specific protein thiols, called reactive cysteine residues, makes H_2_O_2_ an effective second messenger [10].

The cysteine residues of proteins can be divided into four groups based on their reactivity: those that form permanent structural disulfide bonds, those that coordinate with metals, those that remain in the reduced state and those that are susceptible to reversible oxidation. Because the pK_a_ of most protein Cys residues is 8.5 [11], the vast majority of cysteine residues of proteins exist in the reduced state as a Cys-SH at physiological or low pH (pH ≤ 7.2). The cysteine thiolate anion (Cys-S^−^) is readily and reversibly oxidized by H_2_O_2_, whereas the cysteine sulfhydryl group (Cys-SH) is not [12]. Protein cysteine residues can exist as thiolate anions at physiological pH, because the pKa values of these Cys residues are lowered due to the presence of nearby positively charged lysine or arginine amino acid residues. Under these conditions, a pKa of 6.5 is not uncommon. These special thiols are referred to as reactive cysteine residues, and these are specifically targeted by H_2_O_2_. As discussed, these reactive cysteine residues are strategically placed in many proteins and regulate the activity of the protein. The oxidation of reactive cysteine residues frequently results in the formation of an intramolecular disulfide bond with a second protein thiol. However, in the absence of a nearby second thiol, oxidation of the reactive cysteine leads to the formation of sulfenic acid (S-OH), *S*-nitrosothiols (S-NO) or *S*-glutathione (S-SG). These modifications are reversible and can be reduced back to the cysteine sulfhydryl group. Further oxidation leads to the irreversible formation of sulfinic acid (S-O_2_^−^) and sulfonic acid (SO_3_^−2^). Therefore, typically only sulfenic acids further form intra- or inter-molecular disulfide bonds (R-S-S-R) or mixed disulfide bonds with GSH (R-S-SG; called glutathionylation). Thus, the reaction of H_2_O_2_ with a reactive thiol results in the formation of sulfenic acid, which can then react with another Cys in the same or another protein, react with an amide in the backbone of the protein, react with GSH or become further oxidized to sulfinic acid. Interestingly, enzymes, called sulfiredoxins, have been shown to reduce sulfinic acids of certain proteins to sulfenic acids [13].

The primary thiol reductases, Trx and GSH, function as antioxidant proteins, which reduce the oxidized thiol groups of cellular proteins. Trx1 is a 12 kDa protein that regulates redox-sensitive signalling molecules and transcription factors and mediates redox-regulated gene expression. During reduction of target proteins, Trx1 is oxidized and subsequently reduced and regenerated by thioredoxin reductase and NADPH. The Trx1, Trx reductase and NADPH, collectively called the Trx redox system, constitute a key cellular antioxidant system [14]. GSH, a tripeptide (Glu-Cys-Gly), also acts as an antioxidant by forming a mixed disulfide with an oxidized thiol of the target protein. The GSH group is rapidly removed from target proteins by the enzyme glutaredoxin, thereby converting the oxidized cysteine residue of the target protein into a reduced cysteine [15]. In this regard, glutathionylation not only serves to protect protein cysteine residues from irreversible oxidation, but the incorporation of glutathione into the cysteine residue can also regulate protein activity and function. In addition to its role as an antioxidant, GSH also acts as a ROS scavenger. In the presence of the enzyme glutaredoxin, GSH reacts rapidly with H_2_O_2_ to form glutathione disulfide, thereby converting H_2_O_2_ to H_2_O.

Normally, cells utilize transient increases in intracellular ROS levels as a mechanism to activate growth and proliferation. Increased intracellular ROS levels are a hallmark of many cancer cells and are thought to be partially caused by the constitutive activation of the NADPH-dependent oxidases early in the oncogenic conversion of normal cells to cancer cells. For example, oncogenic mutations in RAS genes, one of the most frequent mutations observed in cancer, cause the activation of NADPH oxidases and elevation in ROS levels. The increased ROS levels are tumourigenic by virtue of their ability to maintain cells in a proliferative state and to induce DNA damage, leading to carcinogenesis. Many reports have established that increases in ROS levels are directly correlated with tumour progression, angiogenesis and metastasis [16]. The constitutive oncogenic activation of NADPH oxidases requires that the cancer cell eliminate much of these ROS or risk accumulation of fatal cellular levels. To survive, cancer cells upregulate the proteins involved in ROS detoxification. For example, superoxide anions are converted to H_2_O_2_ and O_2_ by superoxide dismutase, whereas catalase, glutathione peroxidases and peroxiredoxins reduce H_2_O_2_ to water. Interestingly, ionizing or ultraviolet radiation, chemotherapeutic agents, such as etoposide, tamoxifen and doxorubicin, and other agents, such as Cr^6+^, kill cells in part by increasing intracellular levels of ROS.

The essential elements of the GSH/GRx and Trx systems have been identified in the cytoplasmic and nuclear compartments, and it is now clear that cytoplasmic and nuclear redox compartments are independently regulated [15,17–19]. Furthermore, the selective generation of H_2_O_2_ in the nucleus also results in the increased glutathionylation of nuclear proteins [20]. Among the best characterized targets of H_2_O_2_ in the cytosol are PTEN and the protein tyrosine phosphatases (PTPs). H_2_O_2_ specifically oxidizes the catalytic cysteine residue of these enzymes and thereby inhibits their activity [21–23]. Since the activation of receptor protein tyrosine kinases (PTKs) by signalling molecules, such as growth factors, is insufficient to activate the protein tyrosine phosphorylation cascade in cells, because of the overwhelming resting levels of PTP activity, the concurrent inhibition of PTPs by H_2_O_2_ is required by many signal transduction pathways. H_2_O_2_ also targets the Trx-ASK-1(apoptosis activated protein kinase) complex, resulting in its dissociation, movement of ASK-1 kinase into the nucleus, activation of JNK/p38 MAPK and promotion of apoptosis [24,25]. In the nucleus, redox-dependent signalling mechanisms play a critical role in the regulation of gene expression. Many transcription factors, including AP1, Nrf2, NF-κB and p53, possess reversibly oxidizable cysteine residues in their DNA-binding domains, and oxidation of these cysteines blocks their DNA-binding activity [26,27]. Interestingly, redox factor-1 (Ref-1) reduces the oxidized cysteine residue of AP-1 using Trx1 as a reductant. Trx1 translocates into the nucleus in response to H_2_O_2_, tumour necrosis factor and ionizing radiation, where it maintains the critical reactive cysteine residues of transcription factors, such as Ref-1, in a reduced state [17,28].

Recent studies from our laboratory have identified the calcium- and phospholipid-binding protein, annexin A2, as a redox-sensitive protein that played an important role in cellular redox regulation and also functioned on the cell surface as a plasmin reductase. Annexin A2 was also identified by another group as one of nine high-abundance nuclear proteins that was oxidized after increasing nuclear ROS levels [29]. These studies, which have suggested important redox-related functions for annexin A2 on the cell surface, in the cytoplasm and in the nucleus, are discussed below.

## 3. The Annexin Superfamily

More than a thousand proteins in the eukaryotic phyla belong to the annexin superfamily, however annexins are absent from yeast and prokaryotes. Most eukaryotic species have 1–20 annexin genes. Annexins are commonly described as calcium-dependent phospholipid-binding proteins. The classical annexin core domain is made up of four similar repeats of approximately 70 amino acids long (I–IV) (except annexin A6, which has eight repeats). Each repeat is made up of five α helices (A-E), and the type II motif for binding of calcium ions (GxGT-38D/E) is usually located in the loop connecting the A–B helices. In addition to the type II calcium-binding sites, some annexins have type III calcium binding motifs typically located in the loop between helices D and E [30–32]. The *N*-terminus of the annexins is variable and is responsible for distinct localizations, specialized functions and, in some cases, ligand binding. Annexins are mainly localized in the membrane fraction with subpopulations present in the cytoplasm and other subpopulations that are stably or reversibly associated with components of the cytoskeleton. Some annexins bind to other proteins that mediate interactions between the cell and the extracellular matrix. Initially characterized as scaffolding and structural proteins due to their ability to bind to negatively charged phospholipids, increasing evidence has shown that annexins have a multitude of functions and are involved in a wide variety of cellular processes. The theory that annexins have fundamental, non-redundant functions is substantiated by the evolutionary conservation of 12 paralogous genes in humans and the ubiquity of annexins in a wide range of early eukaryotes, including plants and simple organisms, such as *Hydra* and *Geodia* [32].

The first annexin to be attributed to peroxidase activity was the plant annexin from *Arabidopsis thaliana*, AnnAt1 [33–35]. Historically, three studies using heterologous systems established that AnnAt1 could play a protective role during oxidative stress. First, recognition that AnnAt1 could protect cells from oxidative stress came from a study that used a mutant *Escherichia coli* (*E. coli*) strain that lacked the OxyR gene, a transcription factor that senses oxidative stress and is activated by the oxidation of its reactive cysteine residues. OxyR induces the transcription of the regulon of target genes encoding for different antioxidant proteins upon oxidative stress; thus, mutants lacking the oxyR gene are more sensitive to oxidative stress in comparison with wild-type *E. coli.* AnnAt1 was found to restore normal growth of this mutant strain in response to treatment with 350 μM H_2_O_2_. It was suggested that this ability might be due to a catalase-like motif located at the *N*-terminus of AnnAt1, which had a conserved histidine residue (His40) that was predicted to be required for heme-binding. It was also shown that both bacterially-produced and immunoprecipitated AnnAt1 exhibited peroxidase activity *in vitro*. Second, a follow-up paper investigated the capability of AnnAt1 to protect human tumour cells from tumour necrosis factor (TNF)-induced cell death. TNF-α induces the activation of NADPH oxidase and consequent production of H_2_O_2_. Stable transfection of AnnAt1 into a TNF-sensitive HeLa D98 cell line resulted in decreased levels of ROS and resistance to TNF-induced cell death. The authors also found that the transfected HeLa cells showed increased mRNA, protein and activity levels of the ROS-scavenging enzyme, manganese superoxide dismutase, which could explain the ROS buffering effect observed. A third study demonstrated that two different mammalian cell lines transfected with AnnAt1 were protected from H_2_O_2_-induced cell death. The transfected cells showed lower levels of O_2_^•−^, protein kinase C activity and tumourigenicity. Recently, it was shown that partially purified recombinant AnnAt1 contains low levels of peroxidase activity *in vitro* [36]. Nevertheless, this report showed that mutation of the His-40 residue to alanine (His-40-Ala) in the AnnAt1 protein did not block or even inhibit the peroxidase activity [36]. The ability of AnnAt1 to complement the oxyR deletion mutant during oxidative stress was re-tested by this group, and they found that both AnnAt1 wild-type and AnnAt1 His-40-Ala mutant proteins could rescue the *E. coli* oxyR mutant upon H_2_O_2_ insult. All observations showing that AnnAt1 possesses an inherent peroxidase activity are based on assays using AnnAt1 purified with standard chromatography affinity methods to near homogeneity. It is therefore unclear if these protein preparations contain trace amounts of peroxidase(s) or if AnnAt1 actually has peroxidase activity. It is clear though that certain plant annexins provide oxidative protection to cells, as there are numerous cases where ectopic expression or overexpression of an annexin provided transgenic plants with abiotic stress resistance by reducing H_2_O_2_ and/or lipid peroxidation levels [36–40].

It is clear that AnnAt1 is not a classical heme-binding peroxidase [34,36], so a possible alternative mechanism to explain this *in vitro* activity might be one similar to that found for annexin A2 and its binding partner, thioredoxin. Recently, our laboratory showed that annexin A2 functions as a redox regulatory protein [8]. This study provided evidence that the cysteine-8 residue of annexin A2 was selectively oxidized by H_2_O_2_ and then reduced by thioredoxin, thereby freeing the cysteine residue for further redox cycles. Thus, it is possible that AnnAt1 might interact with another antioxidant protein allowing the conserved cysteine residues to function in redox cycles, as we have proposed for annexin A2. Consistent with this possibility, a complex of proteins purified from *Brassica rapa* floral buds that has peroxidase activity contains AnnBr1 and a peroxiredoxin [41]. Recent studies applying a comparative proteomics approach to investigate the potential function(s) of annexin C4 from *A. fumigatus* revealed the downregulation of respiratory chain proteins and the upregulation of a number of stress response proteins in the annxC4 disruptant strain, suggesting the occurrence of a mild oxidative stress phenotype in the annxC4 disruptant strain [42]. This result suggested a possible oxidative stress response function for this fungus annexin.

**Figure 1:**
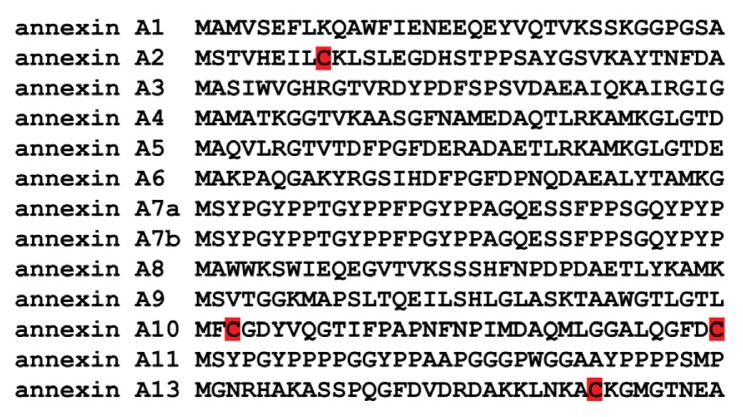
Comparison of the *N*-terminal domain of human annexins. The *N*-terminal domain of the known human annexins were compared using the open source software, Seaview (version 4.3.1) [71]. The *N*-terminal region of the annexins is unique and is responsible for conferring unique functional and regulatory properties to each annexin. The cysteine residues are highlighted in red boxes. The presence of a lysine residue next to a cysteine residue has been shown to lower the pKa of the cysteine residue, thereby converting it into a reactive thiol. The Cys-8 residue of annexin A2 has been shown to be a reactive thiol [8,48,49]. However, experimental work needs to be done to investigate the reactivity of annexin A13 Cys-26 residue.

**Figure 2:**
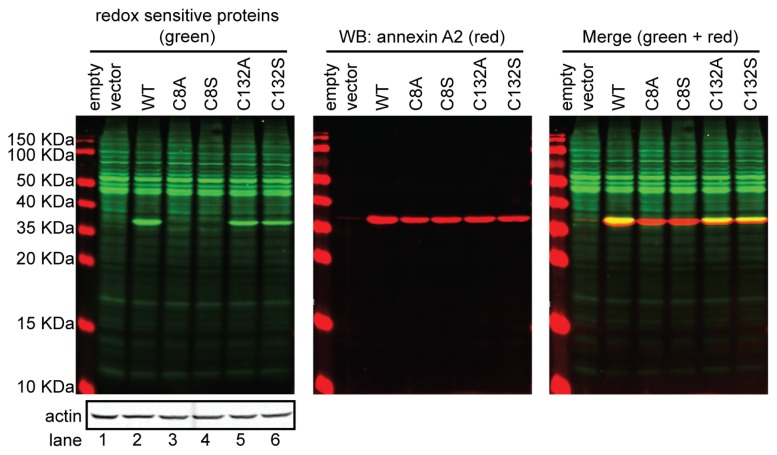
The Cys-8 residue of annexin A2 is a reactive thiol. 293T cells were transiently transfected with pcDNA3 empty vector (lane 1), pcDNA3-ANXA2 WT (lane 2) or a series of ANXA2 cysteine mutant constructs, pcDNA3-ANXA2-Cys-8-Ala (lane 3), pcDNA3-ANXA2-Cys-8-Ser (lane 4), pcDNA3-ANXA2-Cys-132-Ala (lane 5) or pcDNA3-ANXA2-Cys-132-Ser (lane 6), for 48 h. 20 μg of each cell extract was labelled with 20 μM BIAM and subjected to SDS-PAGE, followed by Western blotting with a streptavidin probe (green) and the antibodies indicated: annexin A2 (red) and actin (grey). 293T cells were chosen for these experiments, because of the very low levels of endogenous annexin A2. The data show that mutagenesis of Cys-8 of annexin A2 blocks the labelling of the protein by the reagent, BIAM. Since BIAM labels reactive thiols of proteins, these data establish that Cys-8 of annexin A2 is a reactive thiol.

Annexin A2 can exist in the cells as a monomer or as a heterotetramer (AIIt), where two molecules of annexin A2 are linked by a dimer of the protein S100A10 [30,43,44]. S100A10 is a member of the S100 family of dimeric EF hand-type Ca^2+^-binding proteins. The S100 proteins are small, often acidic polypeptides of approximately 10 kDa, containing an *N*-terminal and a *C*-terminal EF hand separated by a rather unstructured linker region. S100A10 has amino acid mutations in the calcium-binding loops that have rendered these sites unable to bind calcium. However, these mutations have locked S100A10 in a calcium-on conformation. In contrast, all other members of the S100 family are Ca^2+^-regulated [45,46]. Annexin A2 is a planar, curved molecule with opposing convex and concave sides. The convex side of annexin A2 faces the cellular membrane and contains the Ca^2+^- and phospholipid-binding sites, whereas the concave side faces away from the membrane and contains both the *N*- and *C*-terminal regions [30]. The *N*-terminus of annexin A2 contains the binding site for S100A10 [47], a reactive cysteine residue (Cys-8) [8,48,49], phosphorylation sites that are recognized by pp60src (Tyr-23) and protein kinase C (Ser-11, Ser-25) [50,51] and a nuclear export signal (NES) [52], while the *C*-terminal region contains a reactive cysteine residue (Cys-334) [53] and binding sites for F-actin, negatively charged phospholipids, fibrin, RNA and heparin [31,54–59]. Annexin A2 is unique among the vertebrate annexins in that it possesses an *N*-terminal reactive cysteine residue, Cys-8 (Figure 1 and Figure 2). Interestingly, annexin A13, the progenitor annexin for the vertebrate family, may also possess a reactive cysteine, Cys-26, although this has not been confirmed experimentally. Annexin A13 is very limited in its tissue distribution, being expressed only in intestinal and kidney epithelial cells [60]. In contrast, annexin A2 is an abundant and ubiquitously expressed protein in cells and tissues. Annexin A2 is mainly localized in the cytoplasm and plasma membrane [61] with a small, but significant, population located in the nucleus [52,62,63]. Many functions have been attributed to annexin A2. Extracellular membrane associated AIIt was originally identified as a receptor for tenascin-C [64] and as a key plasminogen receptor at the surface of a large number of different cells, including macrophages, endothelial cells and cancer cells [46,65,66]. Cell surface AIIt also functions as a plasmin reductase, promoting the production of the angiostatin fragment, A61 [53]. Cytoplasmic AIIt has been suggested to be involved in endocytic and exocytic vesicular transport, interaction with cell adhesion molecules, regulation of ion channels, mediation of actin dynamics and to function as a scaffold protein in lipid raft organization [67]. One study reported that 15% of cellular annexin A2 was nuclear and was released from the nucleus by RNase A [62]. This is consistent with the report that annexin A2 binds to RNA [68]. Nuclear annexin A2 has also been suggested to play a role as part of a primer recognition protein complex that enhances DNA polymerase activity *in vitro* [69]. We have shown that annexin A2 accumulates in the nucleus in response to ROS-dependent stimulus and mitigates DNA damage [70]. Recently, our laboratory identified a novel function for annexin A2 as a cellular redox regulatory protein [8].

In this paper, we will carry out a comprehensive review of the studies detailing the reactivity of annexin A2 thiols and the importance of these reactive cysteine(s) in regulating annexin A2 structure and function. We will also focus on the recent reports that establish novel functions for annexin A2, namely as a protein reductase and as a cellular redox regulatory protein. We will further discuss the importance of annexin A2 redox regulatory function in disease, with a particular focus on tumour progression.

## 4. Annexin A2 Cysteine Reactivity and Functional Implications

Several studies using a number of different approaches have established that annexin A2 possesses reactive cysteine residues. Historically, the first report that demonstrated the post-translational modification of a cysteine residue of annexin A2 utilized electrospray ionization mass spectrometry to show that Cys-8 residue of annexin A2 could form a disulfide bond with homocysteine *in vitro* [72]. The authors claimed that binding of homocysteine to Cys-8 of annexin A2 interfered with the interaction between tissue-type plasminogen activator (tPA) and annexin A2 [72]. Homocysteine (Hcy) is a thiol-containing amino acid that is an intermediate product in the methionine conservation cycle and in the one-carbon metabolic and trans-sulfuration pathways. 70%–80% of Hcy is bound to protein cysteine residues via a thiol disulfide exchange reaction [73]. The proposed role for Cys-8 of annexin A2 as a binding residue for tPA is controversial. tPA binding to the cell surface has been shown by other laboratories to share a common binding site with plasminogen *in vivo* [46]. Other studies showed that tPA binding to the cell surface was blocked by lysine analogs, such as aminocaproic acid, and was inhibited by 89% by carboxypeptidase B treatment (which removes *C*-terminal lysine residues) [46]. Therefore, a number of studies have supported the concept that both tPA and plasminogen share binding sites on the cell surface and that these binding sites are *C*-terminal lysines of cellular plasminogen receptors. Furthermore, binding of tPA to Cys-8 of annexin A2 through the formation of a disulfide bond contradicts the multiple reports that have rigorously analysed the interaction of tPA with the cell surface and concluded that tPA interacts reversibly with its cell surface receptor(s) [74–76]. A more recent study showed that a number of different peptides containing substitutions of the amino acids in the vicinity of Cys-8 of annexin A2 form a disulfide bond with the annexin A2 Cys-8 residue, but not with tPA [77]. These authors demonstrated that a hexapeptide constituted by amino acids 7–12 of annexin A2, including the Cys-8 residue, did not bind to tPA [77]. This finding further supports that the Cys-8 residue of annexin A2 is a reactive thiol, but not a binding site for tPA.

Another study reported that treatment of AIIt with *N*-ethylmaleimide (NEM), a sulfhydryl reagent that binds to reactive cysteine residues at physiological or low pH (pH ≤ 7.2), resulted in the labelling of AIIt with NEM *in vitro*. Labelling of AIIt with NEM rendered this protein complex unable to mediate liposome aggregation *in vitro.* Nevertheless, NEM-treated AIIt was still able to bind to negatively charged phospholipids [78]. Even though this report showed that AIIt contains reactive cysteine(s), the authors did not investigate which cysteine residue(s) of annexin A2 and/ or S100A10 were modified by NEM.

TNF-α induces the production of ROS by several mechanisms. Many reports have shown that TNF-α signalling upregulates the transcription of various NADPH oxidase components, such as Nox2, Nox3, Nox4, p22phox, p47phox, NOXO1 and p67phox [79–86], which contribute to oxidase activity. Other mechanisms for potentiation of superoxide production by TNF-α have been proposed, such as the phosphorylation of p47phox by downstream kinases, including PKCzeta [87], tyrosine kinases [88–90] and p38 MAPK [91]. TNF-α has also been suggested to regulate NADPH oxidase activity through regulation of the associated hydrogen ion channels [92]. More recently, it was shown that TNF-α is a direct activator of NADPH oxidases in various cell types [93]. The first study that demonstrated the presence of a reactive cysteine residue *in vivo* was reported by Sullivan *et al.*, who identified annexin A2 and the antioxidant protein, thioredoxin peroxidase II, as the major proteins glutathionylated in HT1080 cells upon TNF-α treatment. These studies utilized a novel approach, namely the use of biotinylated glutathione analogue, BioGEE, to tag glutathionylated proteins with biotin [48]. They also found that H_2_O_2_ treatment of HeLa cells led to increased incorporation of the BioGEE label into annexin A2. The authors identified Cys-8 residue of annexin A2 as the target for glutathionylation *in vivo*. In a subsequent study, our laboratory also showed that exposure of HT1080 cells to H_2_O_2_ enhanced the incorporation of BioGEE into annexin A2, further supporting that annexin A2 was *S*-glutathionylated upon oxidative stress [49]. Treatment of AIIt with the thiol-specific oxidant, diamide, resulted in a time- and concentration-dependent loss of the ability of AIIt to aggregate and interact with phospholipid liposomes and F-actin, which was partially reversed by dithiothreitol (DTT), suggesting that AIIt oxidation was responsible for this inhibitory effect. In addition, incubation of AIIt with diamide and GSH resulted in the glutathionylation of AIIt *in vitro*. Mass spectrometry established the incorporation of 2 mol of GSH/mol of annexin A2 subunit at Cys-8 and Cys-132 *in vitro*, whereas the S100A10 subunit of AIIt was not glutathionylated. Glutathionylation of annexin A2 blocked the ability of AIIt to bind to and aggregate liposomes and to interact with and bundle F-actin *in vitro*. We also showed that annexin A2 could undergo functional reactivation by glutaredoxin, which established that annexin A2 can be reversibly glutathionylated [49]. The Sullivan study is in agreement with our more recent results that show that Cys-8 residue of annexin A2 is the reactive cysteine residue *in vivo*, since mutation of Cys-8 to alanine or serine leaves this protein unable to react with the biotinylated iodoacetamide derivative, BIAM [8] (Figure 2). Since the oxidation of the thiolate to a sulfenic acid must occur before a thiol can react with glutathione, Cys-132 may be responsive to oxidation agents, according to the conformation of annexin A2 [15].

Nitric oxide also regulates protein function by covalent attachment of the NO group to cysteine thiolate residues in proteins via *S*-nitrosylation. Liu *et al.* have shown that incubation of AIIt with *S*-nitrosoglutathione (GSNO) led to the inhibition of AIIt-mediated liposome aggregation in a dose-dependent manner [94]. Sodium nitroprusside, another NO donor, also inhibited AIIt-mediated liposome aggregation, whereas GSH, nitrate or nitrite had no effects. Glutathionylation of cysteine residue(s) of annexin A2 has been clearly documented in Sullivan’s and Caplan’s reports; since Liu and colleagues did not investigate if the GSH used reacted with annexin A2, it is difficult to interpret their result showing lack of inhibition of liposome aggregation in GSH treated AIIt samples. GSNO also inhibited AIIt-mediated membrane fusion, but not the binding of AIIt to the membrane [94]. GSNO inhibition of annexin II-mediated liposome aggregation was no longer observed in the presence of dithiothreitol (DTT), suggesting that inhibition of annexin II activity by GSNO was achieved through *S*-transnitrosylation and/or the formation of disulfide bond(s) [94].

## 5. The Role of Annexin A2 As a Plasmin Reductase

Our laboratory showed for the first time that the reactive cysteine residues of AIIt have reductase activity, establishing that these thiols are not only important for the regulation of certain properties and functions of AIIt, but also exert a functional activity of their own within this protein complex [53,95]. The reagent, 3-(*N*-maleimidopropionyl) biocytin (MPB), which reacts with free sulfhydryl containing proteins, was used in order to follow plasmin reduction and AIIt oxidation. Plasminogen and plasmin do not possess free sulfhydryl groups and, for this reason, cannot be labelled by MPB; reduction of plasmin results in the formation of new sulfhydryl groups that can be detected by MPB labelling. Using this approach, we showed that AIIt could reduce plasmin disulfide bonds *in vitro* and that the cysteine residues of annexin A2 and its binding partner, S100A10, participated in the reduction of plasmin. This study established that AIIt and annexin A2 monomer possess intrinsic plasmin reductase activity. AIIt stimulated the reduction of the plasmin Cys-462-Cys-541 bond in a time- and concentration-dependent manner, which resulted in the release of a plasmin fragment (Lys-78-Lys-468), called angiostatin (A61). Mutagenesis of annexin A2 Cys-334-Ser and either S100A10 Cys-61-Ser or S100A10 Cys-82-Ser inactivated the plasmin reductase activity of the isolated subunits of AIIt, suggesting that these cysteine residues directly participated in the plasmin reductase activity of each subunit. Furthermore, loss of AIIt from the cell surface of HT1080 cells transduced with a retroviral vector encoding S100A10 antisense dramatically reduced the cellular production of A61 from plasminogen. This result indicates that AIIt might play a main role not only in plasminogen activation, but also in plasmin inactivation at the cell surface, tightly regulating the activity of this serine protease.

We also investigated the oxidative status of AIIt during plasmin reduction. We showed that incubation of HT1080 cells with plasminogen resulted in the rapid loss of thiol-specific labelling of AIIt by MPB. Since Cys-334 of annexin A2 is not available to the MPB label [53], it is unclear which cysteine residue of annexin A2 becomes oxidized during plasmin reduction. The most likely candidate seems to be Cys-8 of annexin A2, because it is exposed to the solvent and has been shown to react with MPB. In fact, the Cys-8-Ser mutant of annexin A2 cannot be labelled by MPB, indicating that Cys-8 residue of annexin A2 is the only thiol that is available for MPB labelling [53]. Nevertheless, mutation of Cys-8 of annexin A2 revealed that this residue does not directly contribute to annexin A2 plasmin reductase function. Also, treatment of AIIt with iodoacetamide or MPB resulted in a very small decrease in the plasmin reductase activity of AIIt. One explanation for this result is that Cys-8 of annexin A2 might reduce the Cys-334 residue that becomes oxidized by plasmin, thereby resulting in the oxidation of the Cys-8 residue. Another possible explanation is that Cys-334 of annexin A2 is only exposed to the solvent and, consequently, available for MPB labelling after annexin A2/AIIt binding to plasmin. More work needs to be done to investigate these hypotheses.

We further investigated if oxidized AIIt could be subsequently reduced and, in this way, recover its protein reductase function. Several reductants were examined, but only thioredoxin could prevent plasmin dependent oxidation of AIIt, whereas protein-disulfide isomerase, glutathione, cysteine and phosphoglycerate kinase did not reduce AIIt [95]. Future studies are necessary to establish which cysteine residues of AIIt are substrates for thioredoxin reduction. We also performed a comparative study of AIIt with other well-established plasmin reductases. These results showed that AIIt was a more potent plasmin reductase compared to protein disulfide isomerase, thioredoxin and phosphoglycerate kinase under our experimental conditions *in vitro* [53].

**Figure 3:**
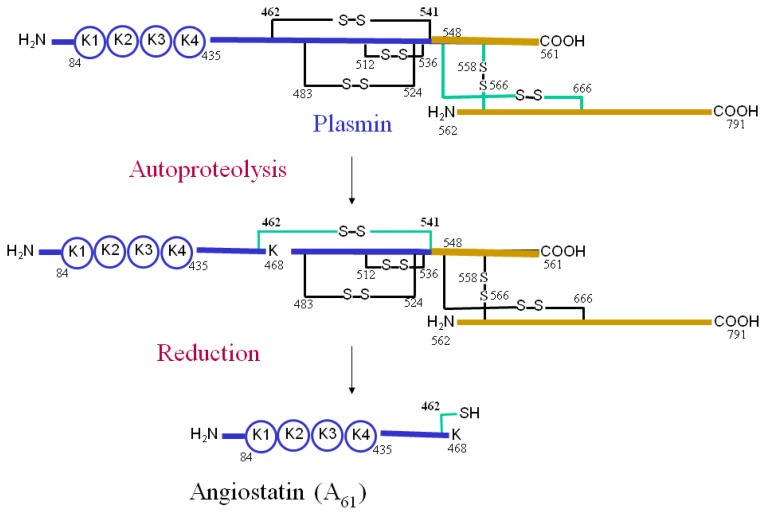
Molecular model for AIIt plasmin reductase function. Plasmin catalyzes the autoproteolysis of the Lys-77-Lys-78 and Lys-468-Gly-469 bonds. AIIt catalyzes the reduction of the Cys-462-Cys-541 disulfide of plasmin, allowing the release of angiostatin, A61.

Taken together, these studies allowed us to propose the following mechanism for the plasmin reductase activity of AIIt (Figure 3). Plasminogen binds to the S100A10 subunit of AIIt and is converted to plasmin by uPA or tPA through the cleavage of the Arg-561-Val-562 bond of plasminogen. Plasmin then catalyzes the autoproteolysis of the Lys-77-Lys-78 and Lys-468-Gly-469 bonds. However, the presence of a Cys-462-Cys-541 disulfide prevents release of angiostatin, A61 (Lys-78-Lys-468), from plasmin. AIIt catalyzes the reduction of the Cys-462-Cys-541 disulfide, which allows the release of A61 from the rest of the molecule. Plasmin autoproteolysis must occur prior to its reduction, since AIIt is not able to reduce catalytically inactive plasmin [53,95]. This suggests that autoproteolyzed plasmin is the substrate for the AIIt. The thioredoxin system then reduces AIIt, enabling it to exert again its protein reductase activity.

More recently, we observed that annexin A2-depleted cells show enhanced oxidation of the annexin A2 binding partner actin and also of the oxidative stress response transcription factor, JunD, upon oxidative damage [8]. These results suggest that annexin A2 and/or AIIt might also function as protein reductase(s) inside the cells. Further studies are necessary in order to investigate this hypothesis.

## 6. The Role of Annexin A2 As a Cellular Redox Regulatory Protein

Recently, our laboratory identified annexin A2 as a novel cellular redox regulatory protein that played a significant protective role in cells undergoing oxidative stress and, in particular, during tumourigenesis.

**Figure 4:**
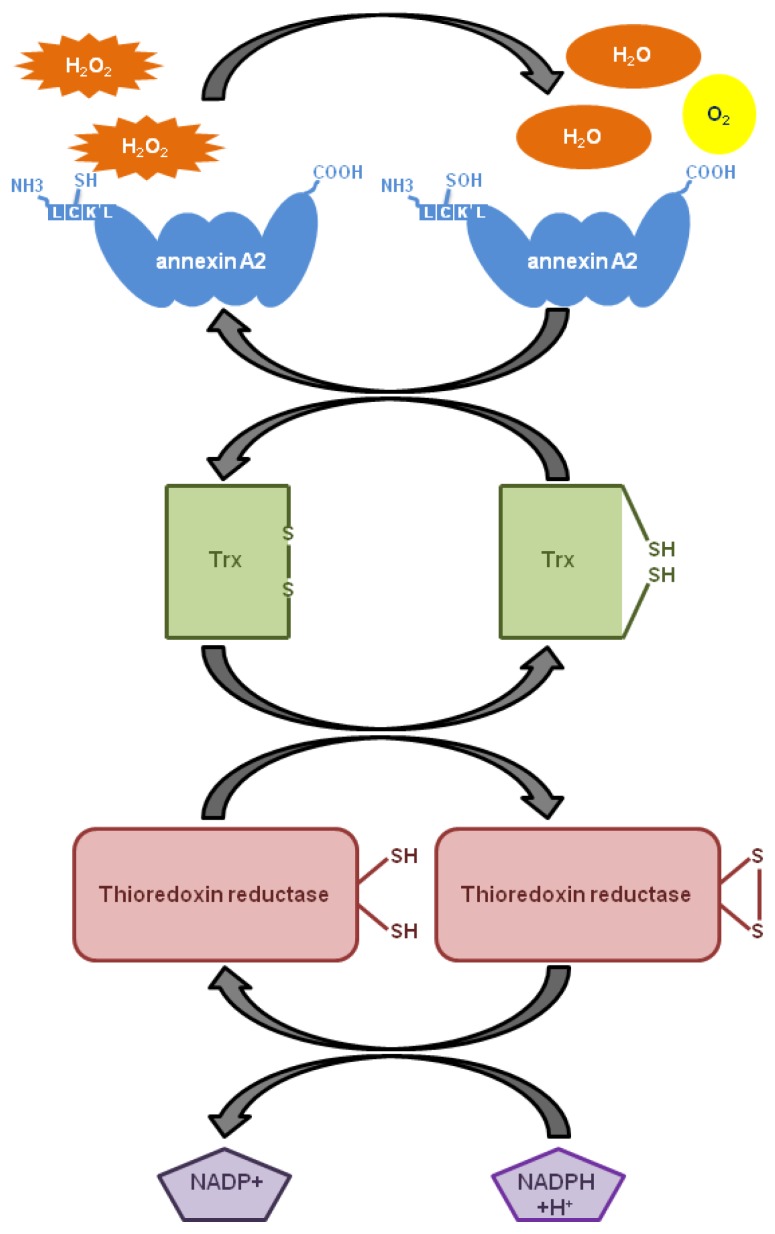
Molecular model for the H_2_O_2_ buffering function of annexin A2. H_2_O_2_ oxidizes Cys-8 of annexin A2, resulting in its conversion to H_2_O and O_2_. Oxidized annexin A2 is then reduced by the Trx redox system and can participate in multiple redox cycles. Thus, a single molecule of annexin A2 can degrade several molecules of H_2_O_2_.

We showed for the first time that Cys-8 residue of annexin A2 is reversibly oxidized by H_2_O_2_ and reduced by the thioredoxin system *in vitro* [8]. Our results allowed us to propose the following mechanistic model: H_2_O_2_ reacts with the Cys-8 of annexin A2, resulting in the oxidation of this residue and the conversion of H_2_O_2_ to H_2_O. Oxidized annexin A2 is then reduced by the Trx redox system and can participate in multiple redox cycles (Figure 4). Thus, a single molecule of annexin A2 has the ability to degrade several molecules of H_2_O_2_.

As mentioned earlier in this review, H_2_O_2_ is an important second messenger in cellular signal transduction that regulates many cellular processes, such as proliferation, differentiation, migration and apoptosis. A tight control in the levels of cellular H_2_O_2_ is then very important for cell signalling regulation and also to avoid protein, lipid and DNA oxidative damage. We showed that annexin A2-depleted cells exhibit significantly higher levels of ROS and increased oxidation of redox sensitive proteins upon treatment with exogenous H_2_O_2_, as compared to control cells. We also verified that depletion of annexin A2 did not alter the expression levels of the main known antioxidant proteins. This result, together with the ability of H_2_O_2_ to directly oxidize the Cys-8 residue of annexin A2, indicated that it is the annexin A2 redox regulatory function that is responsible for the ROS buffering effect observed in oxidative stressed cells. We observed enhanced activation of the pro-apoptotic kinases p38, JNK and Akt in the annexin A2-depleted cancer cells treated with H_2_O_2_ compared to control cells; these protein kinases serve as reporters for the activity of the Ask-1 and PI3K signalling pathways, respectively. The Ask-1 signalling pathway is the major intracellular sensor of oxidative stress induced by H_2_O_2_. Even though the PI3K pathway is usually associated with cell survival, it has been shown that under oxidative stress conditions, Akt further increases the intracellular levels of ROS through an increase in oxygen consumption and inhibition of FoxO transcription factors, thereby sensitizing cells to ROS-mediated apoptosis [96]. Thus, the oxidative stress responsive pathways are more activated in the annexin A2-depleted cells compared to the control cells, most likely due to the higher levels of ROS existent in these cells. This also provides a molecular mechanism that explains the increased sensitivity of the annexin A2-depleted cells to ROS-induced death.

We also showed increased sensitivity of the annexin A2-depleted cells to death induced by chemotherapeutics that increase intracellular ROS levels. Several reports have previously shown that upregulation of annexin A2 in tumours correlates with chemoresistance, but they were unable to explain the mechanism(s) by which this occurs. Our results provided an explanation for this observation, since annexin A2 antioxidant function will protect the cancer cells from oxidative damage/death induced by the chemotherapeutic agents.

The development of an annexin A2 knockout mouse model allowed us to analyse the possible redox function(s) of annexin A2 in the mouse tissues. These experiments showed that annexin A2 knockout mice have enhanced liver and lung protein oxidation compared to wild-type mice. Liver cells have a high metabolic rate, since this organ is involved in protein, lipid and carbohydrate metabolism, synthesis of urea and the manufacture of bile, factors that result in increased ROS production by the mitochondria of the liver. The lung is constantly exposed to oxidative stress due to gas exchange between the alveoli and the red blood cells. Therefore, liver and lung are continually exposed to oxidative stress. Our results showing the presence of high levels of oxidized proteins in the lung and liver tissues of the annexin A2 knockout mouse supported an important cellular redox regulatory role for annexin A2 *in vivo*. However, it was unclear if annexin A2 directly participated in the reduction of oxidized cellular proteins or functioned to prevent the accumulation of ROS in these tissues.

One might expect that if annexin A2 played a key role in cellular redox, the annexin A2 knockout mice should show a more extreme phenotype. However, considering the importance of cellular redox regulation, it is not surprising that cells possess redox proteins with redundant functions. For instance, homozygous catalase knockout mice, which are completely deficient in catalase expression, the major cellular enzyme that decomposes H_2_O_2_, develop normally and show no gross abnormalities under physiological conditions. In fact, mice deficient in catalase were not more vulnerable to hyperoxia-induced lung injury; nor did their lenses show any increased susceptibility to oxidative stress generated by photochemical reaction, suggesting that the antioxidant function of catalase in these two models of oxidative injury is either negligible, which is very unlikely, or is compensated by other cellular antioxidant protein(s) [97]. The only phenotype observed in these knockout mice was a retarded rate in decomposing extracellular H_2_O_2_ by the liver, lung and lens tissue slices compared to the wild-type mice. This phenotype is comparable with what we observed for the annexin A2 knockout mice, even though two different approaches to measure oxidative stress were used.

## 7. Annexin A2 Redox Status and Function in Disease

The importance of annexin A2 redox status and regulatory function in disease remains poorly understood. Recent studies have shown changes in the redox status of annexin A2 under oxidative stress conditions, including cancer. Several proteomic studies that examined changes in the redox status of cellular proteins after treatment with a variety of oxidative stress-inducing agents have identified annexin A2 as a redox sensitive protein.

Jones’s laboratory initially showed that nuclear annexin A2 becomes oxidized in response to oxidative stress [29]. These authors developed a methodology to study oxidation of specific cysteines in high-abundance nuclear proteins. The technique used heavy (^13^C) and light (^12^C) forms of biotin-labelled iodoacetamide to quantify the redox state of nuclear proteins in response to oxidative stress caused by treatment of cells with glucose/glucose oxidase (Glc/Glc oxidase) to generate H_2_O_2_ at a physiological level. The authors isolated the nucleus of these cells and analysed the catalogue of nuclear proteins that responded to oxidative stress. Of the nine high abundance nuclear proteins that were identified in this analysis, annexin A2 showed the most dramatic increase in cysteine oxidation.

Respiratory Syncytial Virus (RSV) infection increases ROS production in epithelial cells by activating NADPH oxidase, while downregulating cytoplasmic catalase and superoxide dismutase [98]. A proteomics study showed a 2.3-fold increase in annexin A2 protein levels in the nucleus of A549 cancer cells infected with RSV compared to uninfected cells [99]. Peroxiredoxin I (PRDX I) and PRDX IV knockdown led to enhanced levels of ROS in the A549 cells and oxidation of cysteine residue(s) in nuclear annexin A2. Unfortunately, the non-nuclear fraction was not analyzed. The authors concluded that the peroxiredoxins protect annexin A2 from oxidation. Nevertheless, it is unclear if annexin A2 and the peroxiredoxins act together in cellular ROS buffering, and depleting the cells from PRDX I and PRDX IV resulted in overwhelming the remaining redox regulatory systems, thereby resulting in the oxidation of annexin A2. Another study showed that peptidyl-prolyl *cis*-*trans* isomerase A, protein disulfide isomerase A(3) (ERp57), serpin B3, annexin A2 and GAPDH were less oxidized in human papillomavirus (HPV)-driven carcinoma samples compared to dysplastic tissues [100]. HPV16 neoplastic progression is associated with an increased oxidant environment. In dysplastic tissues, the oxidative modification of DNA and proteins involved in cell morphogenesis and terminal differentiation may provide the conditions for the neoplastic progression. Conversely, cancer tissues seem to have an improved regulation of oxidative damage, as shown by the selective reduction of carbonyl adducts on key detoxifying/pro-survival proteins. The fact that annexin A2 was one of the five proteins that were less oxidized in the cancer tissues [100] suggests that it might play a main role in ROS detoxification in this cancer type. Nevertheless, this is a hypothesis that needs to be further investigated.

Several studies have also shown an increase in annexin A2 levels upon treatment of cells with exogenous H_2_O_2_- or ROS-producing agents. Ferric nitrilotriacetate (Fe-NTA) induces oxidative renal damage, a consequence of a Fenton-like reaction *in vivo*, leading to a high incidence of renal cell carcinoma (RCC) in rats. Differential display analysis of RCCs showed elevated expression of annexin A2 [101]. Depletion of annexin A2 in Fe-NTA-induced RCC cell lines resulted in apoptosis [101], suggesting that the ROS buffering function of annexin A2 might play an important role in this cancer cell model. The authors showed a time-dependent upregulation of annexin A2 protein and mRNA levels in rat kidney after Fe-NTA administration, as well as in H_2_O_2_ treated LLC-PK1 cells. The later was inhibited by pre-treatment with the antioxidants, NAC, pyrrolidine dithiocarbamate or catalase. It was concluded that annexin A2 expression is regulated by the cellular redox status, but the authors did not investigate if annexin A2 had a redox regulatory function in these cells. Another report used a gel-based proteomics approach in order to investigate if senescent human mesenchymal stem cells (hMSCs) established by incubation with a sub-lethal concentration of H_2_O_2_ (150 μM) are differentially regulated compared to control hMSCs [102]. Annexin A2 and myosin light chain 2 were the only proteins upregulated in oxidative stress-induced senescent hMSCs. The protein and gene expression levels of annexin A2 increased significantly in H_2_O_2_-induced senescent hMSCs compared to control hMSCs, as confirmed by Western blotting and RT-PCR, validating the results obtained by the 2-DE analysis. Our laboratory showed a two-fold increase in annexin A2 levels after treatment of 293T and MCF7 cells with 100 μM H_2_O_2_ [8]. These results suggest that cells might respond to elevations in ROS levels/oxidative stress by inducing the expression of annexin A2.

One study investigated the effect of the Mongolian remedy RuXian-I in the treatment of breast hyperplasia [103]. Rats were either mock treated (control group), injected with estradiol and progesterone to induce breast hyperplasia (model group) or treated with RuXian-I after induction of breast hyperplasia. Subsequently, the authors used an approach that integrated size-based 2D-DIGE, MALDITOF/TOF-MS and bioinformatics to analyze data from the control group, the model group and the RuXian-I-treated group. Fifteen proteins were upregulated, including annexin A2, annexin A1, superoxide dismutase [Mn], peroxiredoxin-1, translationally-controlled tumour protein and α B-crystallin in the model group compared to the control group and downregulated upon treatment with RuXian-I [103]. Multiple biological activities have been reported for the main components of RuXian-I. For example, *Herba Leonuri japonici* has anti-inflammatory and antioxidant activity, suppresses oxidative stress and ameliorates hypercholesterolemia activity. This result suggests that downregulation of ROS levels in the cells decreases annexin A2 protein levels. Nevertheless, this is a very speculative interpretation, since this remedy is composed of several different compounds with diverse properties.

*Ex vivo* experiments using human colon and gastric samples showed that the majority of tumours had enhanced levels of the reduced form of annexin A2 compared to normal tissue, whereas the tumour samples that did not show upregulation of the reduced form of annexin A2 had significantly higher levels of total protein oxidation [8]. These results indicate that annexin A2 antioxidant function is important for the maintenance of the cellular redox equilibrium in human tumours, since, in general, tumours show elevated levels of reduced annexin A2, and loss of reduced annexin A2 led to increased protein oxidation/oxidative stress.

## 8. Annexin A2 Cellular Redox Regulatory Function Supports Tumour Growth

During tumourigenesis, cancer cells typically exhibit enhanced ROS levels. In order to balance the proliferative advantages of ROS upregulation *versus* its potential risks due to protein, lipid and DNA damage, cancer cells induce the overexpression and/or activation of redox regulatory proteins. Recently, we analysed the role played by annexin A2 in tumour growth [8]. We showed that the growth of tumours initiated by the subcutaneous injection of annexin A2-depleted cancer cells was severely impaired compared to tumours initiated with control cancer cells. However, the growth impairment of the annexin A2-depleted tumours could be rescued by the administration of the antioxidant compound, *N*-acetyl cysteine (NAC), in the mice [8]. The tumours formed from annexin A2-depleted cancer cells also showed significantly enhanced protein oxidation (sensor for cellular oxidative damage) compared to tumours initiated with control cancer cells. Nevertheless, there was no significant difference in protein oxidation levels in the annexin A2-depleted tumours compared to control tumours when the mice received NAC [8]. Taken together, these results indicate that annexin A2 depletion resulted in a loss of cellular redox regulatory capability, which became critical as these cancer cells were subjected to enhanced oxidative stress in the tumour site *in vivo*.

It was interesting to observe that NAC by itself stimulated tumour growth of the control cancer cells. This result is consistent with the reports that, compared to normal cells, cancer cells are under significant oxidative stress and that agents that increase cellular ROS levels can selectively kill cancer cells [104,105]. In this situation, NAC can act by lowering the levels of H_2_O_2_ in the tumours, thus increasing cell proliferation or decreasing cell death. This result also indicates that oxidative stress played a major role and is a limiting step in tumour growth under our experimental conditions.

Annexin A2 in its heterotetrameric form, AIIt, has also been implicated in other cellular processes, in particular, plasmin activation, which could also contribute to cancer progression [46]. In this regard, it has been shown that S100A10 acts as the plasminogen receptor, whereas annexin A2 functions to anchor S100A10 to the cell surface [45,46,106]. Several studies have shown that plasmin activation does not play a major role in the subcutaneous growth of primary tumours [107,108]. More importantly, plasmin generation by AIIt or other plasminogen receptors is not regulated by NAC, but is dependent on the presentation of a *C*-terminal lysine residue by the plasminogen receptor [45,95,109]. Consequently, if the growth deficit observed in the annexin A2-depleted tumours was due to decreased plasmin activation, the addition of NAC would not have reversed the phenotype and restored the growth of annexin A2-depleted tumours, as observed.

Several reports have shown that upregulation of annexin A2 levels is positively associated with cancer progression [110] and chemoresistance [111,112]. Nevertheless, the function of annexin A2 during these processes remained unknown. Our laboratory has proposed a molecular mechanism by which annexin A2 contributes to tumourigenesis and resistance to chemotherapy, by acting as a cellular redox regulatory protein. Development of strategies that are aimed at preferentially killing cancer cells through mechanisms that cause additional ROS overload are currently being used. Agents that block annexin A2 expression could therefore be useful to preferentially kill malignant cells.

## 9. Annexin A2 Nuclear Translocation and Protection of DNA from Oxidative Damage

Our laboratory recently showed that genotoxic agents, such as IR, UV-A, etoposide, Cr^6+^ and also H_2_O_2_, promote annexin A2 nuclear accumulation in a ROS-dependent manner, since pre-treatment of cells with NAC prevented the nuclear accumulation of annexin A2. We showed that a GFP fusion protein consisting of the annexin A2 NES fused to the *N*-terminus of GFP (^1^STVHEIL**C**KLSLEGD^15^-GFP) was excluded from the nucleus in mock treated cells, but accumulated in the nucleus in response to H_2_O_2_ [70]. This result suggested that the nuclear accumulation of annexin A2 is most likely regulated by its NES. Since the nuclear accumulation of annexin A2 was blocked by the antioxidant NAC and stimulated by H_2_O_2_, we suspected that oxidation of Cys-8 within the NES might inactivate the NES and promote the nuclear accumulation of annexin A2 by preventing the export of the nuclear protein. However, the GFP fusion protein (^1^STVHEIL**A**KLSLEGD^15^-GFP), consisting of GFP fused to the *N*-terminal region of annexin A2, which had a Cys-8-Ala mutation, was excluded from the nucleus in non-treated cells and also accumulated in the nucleus in response to H_2_O_2_ [70]. This result suggested that the redox status of the Cys-8 residue of annexin A2 does not regulate the NES. We also showed that the annexin A2 binding partner, S100A10, did not accumulate in the nucleus in response to genotoxic agents [70], which was in accordance with a previous study that reported that monomeric annexin A2 accumulated in the nucleus in response to LmB treatment [52].

Other annexins have been shown to accumulate in the nucleus in response to oxidative stress. Plant AnnAt1, as most annexins, does not possess a nuclear localization sequence. However, translocation of AnnAt1 to the nucleus has been observed upon stress stimulation [32,113–116]. Similarly, treatment of cells with H_2_O_2_ has been shown to cause the translocation of mammalian annexin A1 from the cytoplasm to the nucleus [33]. A more recent report has also shown the nuclear translocation of annexin A2 upon treatment of cells with X-radiation [117]. Even though the authors observed increased sensitivity of annexin A2-depleted cells to TNF-α-induced cell death [117], they did not investigate if this was due to annexin A2 cellular redox regulatory function.

The fact that annexin A2 accumulates in the nucleus in response to DNA damaging agents led us to hypothesize that annexin A2 might play a role in protecting DNA from oxidation by ROS. Genotoxic agents are known to directly interact with DNA, causing strand breaks. The rapid phosphorylation of histone H2AX at serine 139 is a sensitive marker for DNA double-strand breaks induced by IR or other genotoxic agents [118]. Interestingly, we observed that treatment of cells with IR or H_2_O_2_ resulted in significantly higher levels of P-H2AX in the annexin A2-depleted cells compared to control cells. The tumour suppressor p53 protein accumulates in the nucleus upon DNA damage, where it functions as a transcription factor regulating cell cycle arrest/apoptosis [119–121]. We observed that annexin A2-depleted cells have increased p53 accumulation upon IR treatment compared to control cells. Several proteins involved in DNA repair and DNA damage signalling, such as phosphorylated histone 2A family member X (γ-H2AX) and the tumour suppressor p53 binding protein 1 (53BP1), have been shown to produce discrete foci that co-localise to DNA strand breaks [122,123]. We showed that annexin A2-depleted cells formed significantly more 53BP1 foci after H_2_O_2_ and IR exposure compared to control cells. These results taken together indicate that annexin A2 plays a role in protecting DNA from genotoxic damage. Since the mechanism(s) of damage and repair for the different genotoxic agents investigated were distinct, the most likely mechanism by which annexin A2 mitigates DNA damage would be through its redox regulatory function. Genotoxic agents, such as IR, can cause oxidative damage to DNA, which results in the formation of 7,8-dihydro-8-oxo-2′-deoxyguanine (8-oxo-G) base mutations in the DNA [124]. We observed that cells depleted of annexin A2 had more oxidative DNA damage than control cells in response to IR, as measured by the presence of 8-oxo-G, supporting the hypothesis that annexin A2 redox regulatory function is important for its ability to protect cellular DNA from oxidative damage.

## 10. Conclusions and Future Directions

The presence of reactive cysteine residues in annexin A2 has been known for many years. However, the recent demonstration by our laboratory of the reversible oxidation of Cys-8 of annexin A2 by H_2_O_2_ and the reversible glutathionylation of this residue has suggested that annexin A2 plays a key role in cellular redox regulation. The exact role that Cys-8 plays in the physiological functions of annexin A2 is unclear, but is under intense investigation. Since extracellular annexin A2 can act as a plasmin reductase, it is possible that annexin A2 may function intracellularly to reduce oxidized proteins. It is also possible that some or all of the functions attributed to annexin A2, such as its role in endocytosis, exocytosis and regulation of ion channels or possibly the transport of dopamine receptors to the cell surface, may be regulated by its redox status. It is also imperative to establish if annexin A2 protein cysteine oxidation by H_2_O_2_ is rapid enough to be relevant for cell signalling. Kinetic studies are necessary to establish the rate at which annexin A2 can detoxify H_2_O_2_ and evaluate its role in the direct regulation of H_2_O_2_ levels in the cells under normal conditions or during oxidative stress. Therefore, future studies will be necessary to establish if the reactive cysteine residue(s) of annexin A2 function as a peroxidase by directly interacting with ROS or by acting as a redox sensor and regulating other proteins and/or peroxidases. The reactive cysteines of annexin A2 could function to regulate the interaction of annexin A2 with other cellular structures, such as the lipid rafts or the cytoskeleton. Previous studies demonstrating the role of annexin A2 in processes, including exocytosis, endocytosis, the regulation of the cytoskeleton and membrane organization and ion conductance, coupled with our studies identifying annexin A2 as a redox protein, open up many exciting possibilities for the function of this pleiotropic protein.
